# Three-Dimensional Neurophenotyping of Adult Zebrafish Behavior

**DOI:** 10.1371/journal.pone.0017597

**Published:** 2011-03-07

**Authors:** Jonathan Cachat, Adam Stewart, Eli Utterback, Peter Hart, Siddharth Gaikwad, Keith Wong, Evan Kyzar, Nadine Wu, Allan V. Kalueff

**Affiliations:** Department of Pharmacology and Neuroscience Program, Tulane Neurophenotyping Platform and Zebrafish Neuroscience Research Consortium, Tulane University Medical School, New Orleans, Louisiana, United States of America; University of Minnesota, United States of America

## Abstract

The use of adult zebrafish (Danio rerio) in neurobehavioral research is rapidly expanding. The present large-scale study applied the newest video-tracking and data-mining technologies to further examine zebrafish anxiety-like phenotypes. Here, we generated temporal and spatial three-dimensional (3D) reconstructions of zebrafish locomotion, globally assessed behavioral profiles evoked by several anxiogenic and anxiolytic manipulations, mapped individual endpoints to 3D reconstructions, and performed cluster analysis to reconfirm behavioral correlates of high- and low-anxiety states. The application of 3D swim path reconstructions consolidates behavioral data (while increasing data density) and provides a novel way to examine and represent zebrafish behavior. It also enables rapid optimization of video tracking settings to improve quantification of automated parameters, and suggests that spatiotemporal organization of zebrafish swimming activity can be affected by various experimental manipulations in a manner predicted by their anxiolytic or anxiogenic nature. Our approach markedly enhances the power of zebrafish behavioral analyses, providing innovative framework for high-throughput 3D phenotyping of adult zebrafish behavior.

## Introduction

Mounting evidence demonstrates the utility of zebrafish (*Danio rerio*) in neurobehavioral research [1], [2], [3], [4], [5]. As a vertebrate species, they share substantial genetic and physiological homology with mammals, possessing all major neurotransmitters, hormones and receptors [6], [7], [8], [9], [10], [11], [12]. Soon after birth, zebrafish begin to display basic locomotory behavior [13], [14]. As a result, behavioral assays monitoring multiple larvae *in parallel* are widely used as high-throughput screens for genetic research and drug discovery [15], [16], [17], [18]. The strength of larval models is in their high-throughput nature, ease of genetic manipulations, and simple, well-defined behavioral endpoints [19], [20], [21], [22]. However, adult zebrafish exhibit complex behaviors (e.g., social [23], [24], learning [25], [26] and affective responses [27], [28], [29]) offering a unique translational opportunity to model brain disorders [4], [9], [12], [30]. The strengths of adult zebrafish models include the relevance of adult fish physiology to human brain disorders; well-developed motor, sensory and endocrine systems; high sensitivity to environmental challenges, and a wide spectrum of behavioral phenotypes [1], [2], [5], [28], [31], [32], [33], [34].

**Figure 1:**
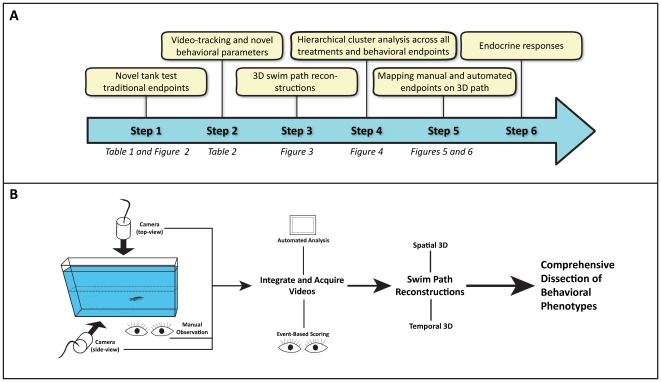
Flowchart illustrating the experimental strategy of this study. The rationale (A) includes examining traditional, manually recorded novel tank test behavioral endpoints across several treatments and trials (Step 1). Video-tracking analysis was then performed to generate additional automated behavioral endpoints and raw spatiotemporal data for three-dimensional (3D) swim path reconstructions (Steps 2–3), followed by hierarchical clustering (Step 4) across all behavioral endpoints and experimental treatments in order to discover potential overlaps between manual and automated endpoints. These overlaps were reconfirmed using the 3D swim path reconstructions (Step 5). Finally, our interpretation of the observed affective states was verified with measured endocrine responses (Step 6). The experimental process (B) was standardized for all novel tank trials. Naïve, wild-type zebrafish were placed in an unfamiliar, novel tank for 6 min. Animal behavior was manually observed and two cameras recorded videos for automated analysis in EthoVision XT7 (during which manual, event-based scoring was also performed). Track data for each subject was exported, processed and visualized in a 3D scatter plot with RapidMiner 5.0.

**Figure 2:**
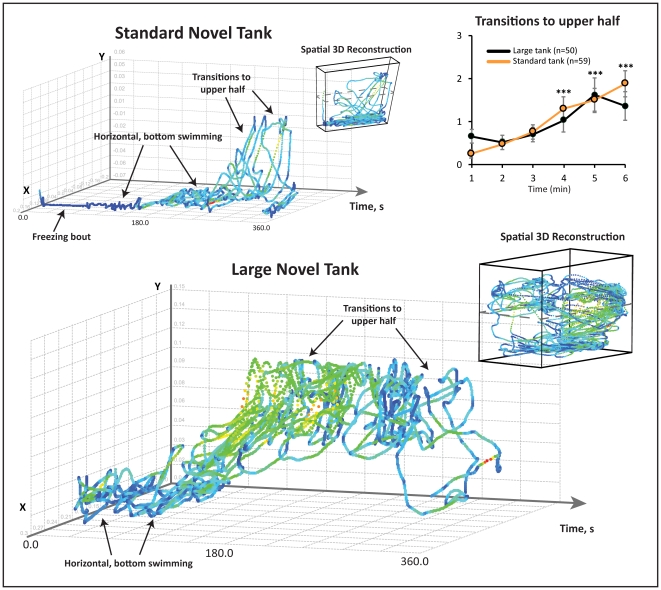
Exploratory behavior of adult zebrafish in two different novel tank apparatuses. Temporal three-dimensional (3D) reconstructions plotted X,Y-coordinates (exported from EthoVision XT7 video-tracking software) on respective X,Y-axes, with experimental time plotted across the Z-axis (see Fig. 4 for an example). Spatial 3D reconstructions were generated in a similar fashion, with spatial coordinates from a top-view recording plotted on the Z-axis (see Fig. 5 for an example). Arrows indicate swimming activity patterns of interest; note the overall similarity of behavioral dynamics across two different novel tanks. Track color reflects changes in velocity (m/s), moving from dark to light (i.e., from blue to green, yellow and red) as velocity increases. Zebrafish placed in standard (small) or large novel tank displayed similar exploratory behavior dynamics (also see transitions to top as an example). Two-way ANOVA (factors: tank type; test time) revealed no tank type effect across all manual endpoints, but a significant time effect with transitions to and time spent in the upper half, increasing and freezing bouts and duration decreasing over time (F_(1,5)_ = 2.1-9.3, p<0.05; ***p<0.01, post-hoc test vs. the respective min 1). This figure serves two purposes. First, it illustrates that the approach presented here can be applied to novel tanks of various shapes and sizes. Additionally, it validates the small novel tank test as a paradigm suitable for standardized phenotyping of zebrafish anxiety-like behavior.

**Figure 3:**
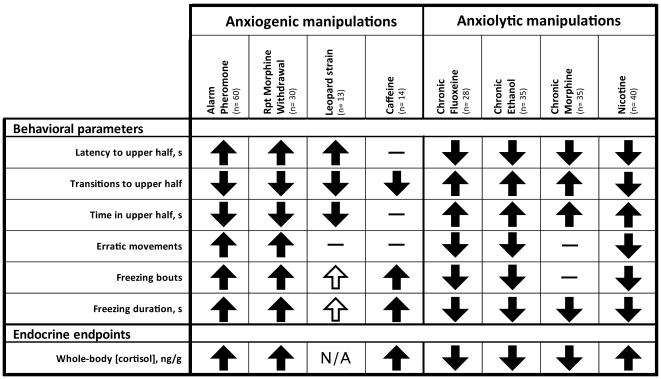
Summary of anxiogenic and anxiolytic modulation of adult zebrafish behavior in standard 6-min novel tank test (see Fig. S1 in Supplementary Materials for raw data from these experiments). This analysis illustrates the strong predictive validity of traditional, manually quantified behavioral endpoints in the novel tank test. Well-established ‘reference’ anxiogenic manipulations include acute alarm pheromone (7 mL for 5 min), repeated morphine withdrawal (exposure for 1.5 mg/L for 2 weeks; withdrawal for 3 h twice daily for 1 week), acute caffeine (250 mg/L for 20 min) and the use of high-anxiety leopard zebrafish strain. Reference anxiolytic treatments include chronic fluoxetine (100 µg/L for 2 weeks), chronic ethanol (0.3% vol/vol for 1 week), chronic morphine (1.5 mg/L for 2 weeks) and acute nicotine (10 mg/L for 5 min). Statistically significant differences from matched controls (p<0.05, U-test) are represented by solid arrows (empty arrows denote trends; p = 0.05–0.085, U-test): up – increase, down – decrease; empty fields indicate no significant effects, n/a – data not available. In addition to behavioral endpoints, the table also includes whole-body cortisol data, presented here to parallel zebrafish behavioral and physiological anxiety-like responses.

Recent studies have characterized adult zebrafish behavior in several novelty-based paradigms, reporting habituation [35], thigmotaxis, geotaxis and scototaxis [28], [34], [36], [37]. As a relatively young field, adult zebrafish behavioral neuroscience continues to adapt traditional rodent paradigms (such as open field, light-dark box, startle, and predator exposure tests) to the use in this aquatic species [4], [5], [31], [37], [38], [39], [40], [41]. Similar to rodent open field test [42], [43], [44], the novel tank test (Fig. 1 and 2) evaluates the natural neophobic response of zebrafish, expressed in reduced exploration, increased freezing and/or unorganized erratic locomotion [27], [28], [35], [39], [45]. In contrast, reduced anxiety in this test is accompanied by increased exploration with reduced freezing and fewer erratic bouts [28], [46] (Fig. 3).

While manual registration of behavior is time-consuming and prone to subjective variation [13], [47], [48], video-tracking technology helps rapid and objective quantification of zebrafish behavior [47], [48], [49]. These tools also provide additional basic indices (i.e., distance traveled, velocity) and more complex parameters (i.e., body elongation, angular velocity) which cannot be scored manually (see Table S1 in Supporting Information for details) [50]. Video-tracking has been broadly applied to fish research [51], [52], [53], [54], [55] (including zebrafish [34], [39], [48], [56], [57], [58], [59]), focusing on swimming mechanics [60], [61], [62], [63], [64], [65] and detection of multiple subjects in shoaling studies [24], [59], [66], [67], [68]. Previous attempts to integrate manual and automated anxiety-related endpoints (e.g., [56]) were limited by the lack of sophisticated software (able to integrate data) and using lower sampling rates (unable to generate enough data points). Other reports have either applied 2D (one camera) video-tracking methods to assess fish stress-related behaviors [69], [70], [71], or used 3D (two cameras) video-tracking [72] as well as high sampling rate to characterize fish swimming, including assessment of zebrafish neurotoxic phenotypes [73], [74] (also see [75]). Respectively, there were no published studies that applied 3D camera set-ups to characterize zebrafish anxiety-related behavioral phenotypes, in which manual and automated data were precisely integrated and acquired using the maximum sampling rate.

Several factors were critical for our research strategy (Fig. 1). First, we used the latest version of EthoVision XT7 software (Noldus IT, Wageningen, Netherlands) with manual event-based scoring during the video acquisition. This allowed us to precisely integrate manual and automated endpoints into a single track file, overcoming the methodological challenges of earlier zebrafish studies [56]. Secondly, by acquiring videos at the maximum sampling rate, we markedly increased the data density for each subject with raw track files containing spatiotemporal, movement and manual data points for every 0.033 s. Upon this realization, we attempted to reconstruct swim paths using this rich spatiotemporal information. We applied data-mining software (Rapid Miner 5.0, Rapid-I GmbH, Dortmund, Germany) that became available only recently, to visualize zebrafish swim paths. To comprehensively dissect adult zebrafish behavior, we compared the effects of multiple anxiogenic and anxiolytic experimental manipulations (Fig. 3), created three-dimensional (3D) reconstructions of swim paths (Fig. 2, 4–6) and performed hierarchical cluster analyses (Fig. 7). Taken together, these strategies enabled us to 1) improve data handling by consolidating raw data, 2) rapidly examine overall zebrafish behaviors, and 3) optimize video-tracking settings to more accurately detect the behaviors of interest. Furthermore, compared to traditional bar/line graphs or 2D traces, these 3D reconstructions provide intuitive representation of zebrafish activity which can be used for global evaluations and visualization of observed affective states (Fig. 6).

**Figure 4 pone-0017597-g004:**
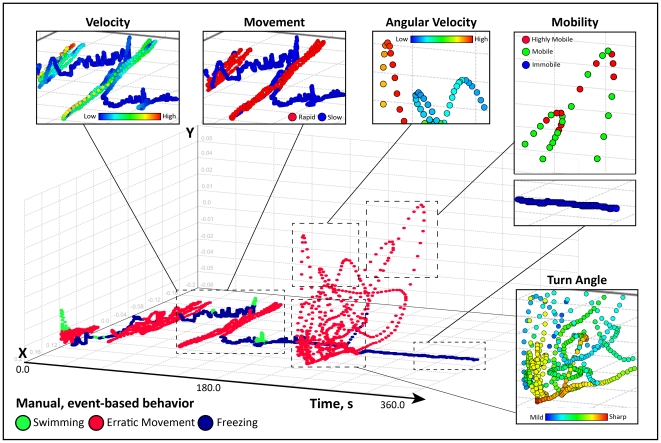
Dissection of zebrafish behavior using three-dimensional (3D) temporal reconstruction of swim path. The reconstructed swim path was obtained from a zebrafish tested in a standard novel tank test (Fig. 1) for 6 min following repeated morphine withdrawal, an anxiogenic manipulation (Fig. 3). Manual, event-based behaviors were scored by a human observer during automated video acquisition in EthoVision XT7 program. Subsequently, spatiotemporal (X,Y,time) coordinates, computer-generated movement parameters and manually-scored behaviors were integrated into a single track file for each subject using RapidMiner 5.0 software. After X,Y-coordinates were plotted over experimental time, behavioral endpoints were actively cycled across the swim path as the color attribute and examined for overlaps and patterns. The experimental manipulation used here caused long, prominent freezing bouts separated by short bursts of bottom swimming – a profile typically observed in zebrafish high-anxiety states (Fig. 3). A detailed dissection reveals that manually scored erratic movements on a 3D reconstruction map within episodes of elevated velocity, rapid movement, high angular velocity, high mobility and sharp turn angles (generated by the computer). Conversely, manually scored periods of freezing correlate with lower velocity, slow movement and immobility bouts. For better visuality, the observed endpoints were color-coded, with the legend color scales representing proportional spectrum across min/max ranges of observed experimental values. This experiment shows that 3D temporal reconstructions permit rapid and objective macro- and micro-level behavioral analysis, thereby improving high-throughput phenotyping of zebrafish behavior. This method of multidimensional phenotyping of zebrafish locomotion can complement spatial 3D reconstructions (as shown in Fig. 5 and 6).

**Figure 5 pone-0017597-g005:**
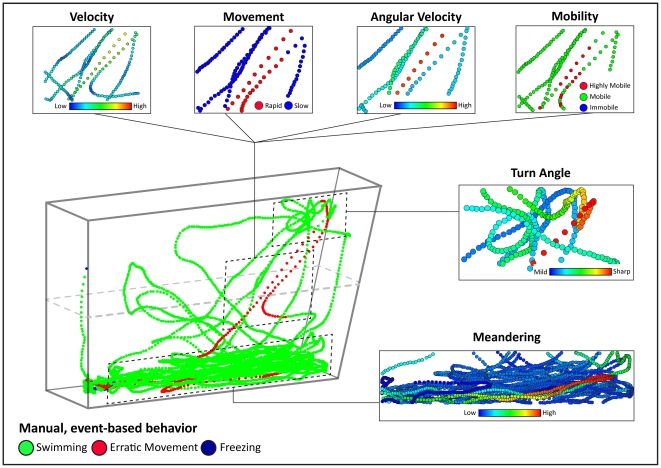
Macro- and micro-level behavioral analysis with three-dimensional (3D) spatial reconstruction of swim path. The reconstructed swim path presented here as an example was obtained from a naïve, wild-type control zebrafish tested in a standard novel tank test for 6 min (see Fig. 6 for more examples). Wild-type fish can be considered “mild anxiety”, compared to both anxiolytic (low anxiety) and anxiogenic (high anxiety) cohorts listed in Fig. 3. Although this fish spent a majority of the trial within the bottom half of the tank, the animal also made large sweeping transitions into the upper half. A detailed spatial dissection of 3D locomotion here revealed that (like temporal 3D reconstructions in Fig. 4) manually scored erratic movement events generally overlap with periods of elevated velocity, rapid movement, high angular velocity, high mobility and sharp turn angles, identified by the computer analysis. For better visuality, the observed endpoints were color-coded, with the legend color scales representing proportional spectrum across min/max ranges of observed experimental values. Overall, this approach strongly supports the utility of 3D-based computer-aided analyses of zebrafish behavior, and for the first time creates 3D reconstructions of zebrafish *natural* exploratory locomotion, mapping anxiety-related behaviors to these traces. The striking overlap between observer- and computer-generated indices in “real” 3D traces open opportunities for further refinement of video-tracking, eventually leading to fully automated 3D-based neurophenotyping tools to quantify zebrafish anxiety. This method of multidimensional phenotyping of zebrafish locomotion can complement temporal 3D reconstructions (as shown in Fig. 4 and 5).

**Figure 6 pone-0017597-g006:**
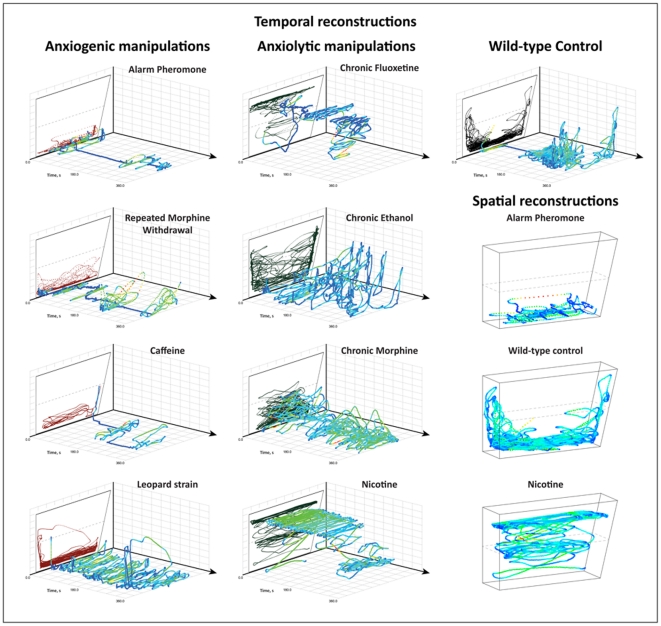
Three-dimensional (3D) temporal and spatial reconstructions of adult zebrafish swim paths rapidly expose overall affective phenotype. After indicated experimental manipulations (Fig. 3), zebrafish novel tank behavior was manually observed and video-tracked using EthoVision XT7 program. Raw track and behavioral endpoints were processed, formatted, and visualized in a 3D scatter plot using RapidMiner 5.0 software; traditional computer-generated two-dimensional (2D) swim path traces were placed at t = 0.0 s (top left part of each panel) for reference. Representative 3D reconstructions were selected by comparing swim paths of all subjects within a cohort, ranking them from 1 to n based on similarity to each other (low/no to high activity) and choosing the middle for the illustrations. For better visuality and consistency, fish used for spatial 3D imaging were the same as those used for the respective temporal 3D reconstructions. For a more detailed analysis of 3D reconstructions, the average velocity (m/s) of each fish was reflected by changes in color from blue to green, yellow and red, as the velocity increases. Note that any other computer-generated behavioral indices (Table S1 of Supporting Information) may be expressed in color on 3D reconstructions of zebrafish locomotion paths. Overall, these 3D traces reveal striking differences between zebrafish high- and low-anxiety behaviors, thereby enabling a rapid visualization and interpretation of the observed phenotypes.

**Figure 7:**
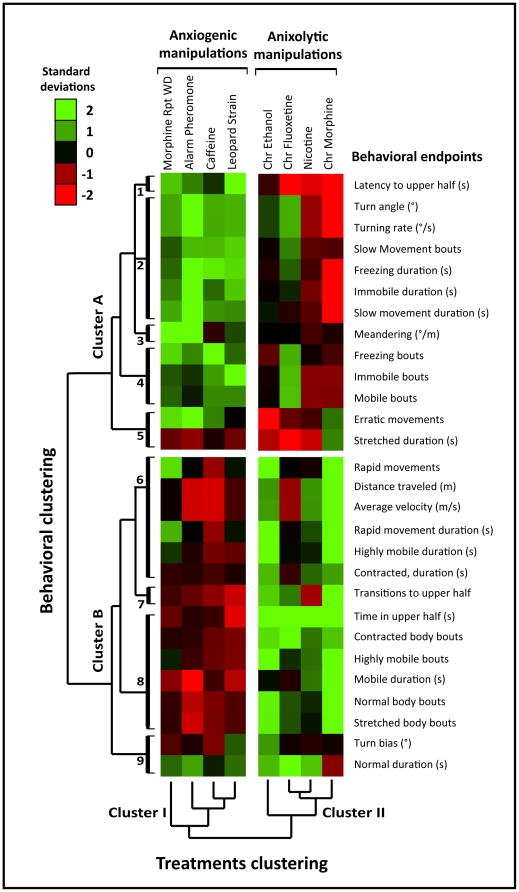
Hierarchical cluster analysis (performed for the entire 6-min test duration) groups anxiogenic and anxiolytic manipulations and correlates manual and computer-generated behavioral endpoints. Experimental manipulations were hierarchically clustered to link compounds to behaviors (based on behavioral endpoints listed in Table S1 of Supporting Information, generated using the side-view video-tracking by EthoVision XT7). In the clustergram, each cell represents the average relative value in standard deviations (green – higher, red - lower than controls) for each behavioral endpoint. Clustering of all 8 treatments (Fig. 3) resulted in two main clusters (I and II) strikingly corresponding to known anxiogenic and anxiolytic manipulations (Rpt WD - repeated withdrawal, Chr - chronic treatment). For behavioral clustering, dark bars (labeled 1–9) represent specific sub-clusters analyzed in detail for manual and automated endpoints organized in two main clusters (A and B). Note that anxiogenic manipulations (Fig. 3; cluster I) generally reduce the cluster A endpoints and increase the cluster B indices. Anxiolytic treatments (cluster II) demonstrate the opposite phenotype, increasing the cluster A behaviors and reducing cluster B endpoints. Overall, this analysis not only reconfirms the validity of traditional novel tank endpoints and manipulations, but also identifies some novel computer-generated endpoints that reflect zebrafish high- and low-anxiety states. Notably, some of these novel indices already demonstrated sensitivity to anxiety-like states, as illustrated by three-dimensional (3D) reconstructions in Fig. 4–6.

Spatiotemporal 3D swim path reconstructions have several important advantages over traditional 2D traces or time series plots. First, they provide a more “realistic” (i.e., 3D vs. 2D) representation of zebrafish swimming activity, minimize potential errors in interpreting fish lateral movements (towards the camera/observer), and simplify comparison of manual, event-based and automated endpoints for each fish within a single track file. 3D reconstructions also represent valuable visualizations of general behavioral patterns, and therefore can facilitate sharing and communicating experimental results.

Overall, this study sought to conceptually advance neurophenotyping of adult zebrafish by integrating manual observation and automated acquisition of behavioral parameters while seeking naturalistic, more relevant 3D representations of zebrafish novelty evoked behavior as it occurs. Accordingly, the aim of our study was not to develop a new phenotyping software, but to show that integrated assessment of spatial, temporal and movement behavioral endpoints has a strong potential to improve the characterization and interpretation of complex anxiety-related adult zebrafish behavior.

## Results

We first examined the exploratory behavior of naïve, wild-type zebrafish in either a standard (small) or large novel tank (Fig. 2). The fish progressively displayed significantly more transitions to and time in the upper half, while showing fewer freezing bouts and shorter freezing duration over the 6 min testing period, regardless of the tank size. To illustrate the common dynamics of this behavior, temporal and spatial 3D swim path reconstructions were generated for both tanks, again showing overt similarities in zebrafish exploration strategies (see Fig. 2 for details). This figure shows how the representative zebrafish made short, horizontal sweeps, remaining low in the novel tank for approximately half of the test, before making several large and smooth swipes, exploring upper levels of the novel tank approximately every 30 s).

Using the novel tank test as a standardized validated paradigm, we next tested the effects of known anxiogenic and anxiolytic manipulations on manually scored zebrafish behavior (Fig. 3, also see Fig. S1 in Supplementary Materials). Consistent with the first experiment, anxiogenic manipulations here predictably decreased top exploration while increasing freezing activity and erratic movements. In contrast, anxiolytic manipulations resulted in a reduction of erratic and freezing behavior, while increasing exploration in the upper half of the novel tank (Fig. 3). Therefore, these experiments support the novel tank test as a model of stress-evoked adult zebrafish behavior, reproducible across different experimental manipulations.

After assessing the traditional manually-recorded parameters (Fig. 3), we investigated the potential of video-tracking software to further dissect zebrafish behavior. Temporal 3D reconstructions plot X,Y- coordinates and time within a 3D scatter plot, resulting in an interactive depiction of zebrafish swimming activity. As shown in Fig. 4, a representative anxiogenic treatment - repeated morphine withdrawal - revealed a typical anxiety-like response (observed in many other fish of this group), suggesting the value of temporal 3D reconstructions in dissecting zebrafish anxiety-like behavior. Notice several high-velocity, short-lived bursts of horizontal sweeps along the bottom edge of the novel tank, which were separated by extended bouts of bottom freezing. After more than half of the trial has past, a freezing bout was interrupted by several high-speed swipes towards (and rarely into) the upper half. This swimming profile is strikingly different from naïve wild-type zebrafish, and is clearly anxiogenic because these horizontal sweeps occur in rapid succession (rather than having distinctive or regular intervals between upper exploratory sweeps). Acquiring videos at the maximal sampling rate also unexpectedly resulted in “noise” at times when the zebrafish remained motionless, freezing at the bottom of the tank. Recognized and evidently illustrated in swim path reconstructions (notice small bounces or hills during periods the fish is manually registered as freezing), this “noise” is a result of rapid flips in the subject center-point detection during automated video acquisition. Upon review, automated endpoints which are calculated over a change in distance (e.g., meandering) were also effected by this acquisition “noise”. Fortunately, 3D swim path reconstructions provide a means to quickly examine several small changes in video tracking settings and optimize subject detection to reduce these issues (discussed in detail below).

Manually scored behaviors in this experiment were attributed across the 3D reconstruction to compare automated parameters with known behavioral states (also see Table S2 in Supporting Information). We found that manually recorded erratic movements correspond to episodes of increased velocity, highly mobile bouts, high angular velocity (turning rate) and sharp turn angles. Conversely, freezing bouts (recorded manually) corresponded to episodes of slow movement, immobile bouts and decreased velocity, detected by computer on 3D reconstructions (Fig. 4). Spatial 3D reconstructions integrate spatial coordinates from two cameras (positioned as in Fig. 1) within an X,Y,Z-scatter plot (Fig. 5), again showing how video-tracking software detects complex anxiety-like behaviors previously limited to manual quantification (see Fig. 6 for other generated 3D traces, and Supplementary Material for details). In Fig. 5, notice the smooth, widely distributed exploratory sweeps to the upper half, and the overlap in manually-scored erratic movements with bouts of rapid movement, high mobility, increased angular velocity and sharper turn angles. In conclusion, the advantages of 3D reconstructions include 1) consolidation of manual, automated and spatiotemporal parameters into a single data file; 2) rapid examination of overall behavioral states; 3) detection of potential abnormalities in subject detection, and 4) a way to quickly examine a variety of video-tracking settings to optimize quantification of zebrafish swimming activity.

In order to reconfirm observed correlations between experimental manipulations and behavioral endpoints, a hierarchical cluster analysis was performed (Fig. 7). This unsupervised procedure identified several interesting patterns, with experimental manipulations clustering within two distinct groups (I and II). This analysis was limited to large-scale similarities in the magnitude of the endpoints' deviation from matched experimental controls. Additional validation, particularly for automated endpoints such as body elongation, will be necessary to fully understand how experimental treatments change these parameters. Cluster I consisted of repeated morphine withdrawal, alarm pheromone, caffeine, and the leopard strain, all known to evoke high-anxiety states. Cluster II included chronic ethanol, morphine, fluoxetine, and acute nicotine all corresponding to anxiolytic-like treatments. Importantly, this unsupervised procedure accurately distinguished between known anxiogenic and anxiolytic treatments (Fig. 3) based on a global analysis of zebrafish phenotypes.

We next examined clustering of all behavioral endpoints recorded here. Traditional and automated parameters clustered into two major groups (clusters A and B), with strongly correlated endpoints forming multiple sub-clusters (Fig. 7). To simplify the clustering, we reduced these sets to distinct sub-clusters using a correlation coefficient ≥0.5 as a cut off criterion. The first cluster (cluster A) consisted of 5 sub-clusters. Sub-cluster 1 contained latency to top (a primary novel tank behavior measure from Fig. 3), reflecting the well-established fact that anxiogenic treatments generally increase, and anxiolytic treatments decrease, this index in both manual and video-tracking analysis. Sub-cluster 2 included turn angle and rate, slow movement frequency and duration, as well as freezing and immobility duration, which were all similarly increased by anxiogenic treatments. This clustering also highlighted a strong correlation between freezing duration (manual) with two distinct approaches to automatically quantify freezing - slow movement and immobility duration, an overlap suggested to occur in 3D swim path reconstructions, as noted above. Sub-cluster 3 represented meandering, indicating that this endpoint is sensitive to anxiogenic challenges, and is generally attenuated by anxiolytics. Freezing bouts, immobile bouts and mobile bouts were grouped within sub-cluster 4, providing a second instance in which manual and automated quantification of the same behavior (e.g., freezing frequency) were highly correlated, as hypothesized after examination of 3D swim path reconstructions. Sub-cluster 5 contained erratic movements and stretched duration. Overall, Cluster A contained behavioral endpoints that are prominently expressed in high-anxiety states, and are generally reduced by anxiolytic manipulations (Fig. 7). However, the specific relationship of these new automated endpoints will require further validation before their full potential is realized. It is expected that the techniques presented in this manuscript will strongly facilitate these efforts.

The second major group (Cluster B) included 4 sub-clusters. Sub-cluster 6 combined mainly locomotor endpoints, such as rapid movements frequency and duration, distance traveled, average velocity, rapid and highly mobile movement durations, and contracted swimming duration. Sub-cluster 7 included transitions to the upper half, a primary novel tank test endpoint increased by anxiolytic treatments. Time in the upper half, strongly increased by anxiolytic factors, was grouped within sub-cluster 8, together with contracted body bouts, high mobility bouts, mobile duration, normal body swimming, and stretched body shape. Sub-cluster 9 contained turn bias and normal body duration, both of which (unlike all previous endpoints) showed little variation between treatment groups (Fig. 7).

To determine the reliability of manual and automated behavioral quantification here, Spearman correlation coefficients for primary endpoints were calculated between manual observation (performed during the novel tank test), event-based scoring (performed by trained observers during acquisition) and automated video-tracking of zebrafish movement (see above). Table S2 of Supporting Information compares manual observation with automated quantification of traditional novel tank endpoints, such as latency and transitions to, and time spent in the upper half. A significant correlation was found across several experimental trials (Table S3 of Supporting Information), illustrating the reliability of automated video-tracking tools. The reliability of video tracking to accurately quantify more complex behavioral states such as erratic movements and freezing was also examined (see Fig. S2 in Supporting Information for details). Based on the observed overlap in 3D reconstructions, we compared automated detection of rapid movements and highly mobile bouts to manual scoring of erratic movements. Likewise, automated parameters for slow movement, immobility and not moving were evaluated against manually scored freezing bouts and duration. In both cases, default video tracking settings reported endpoint values that were highly over-estimated (see default settings in Fig. S2 of Supporting Information). However, after optimization procedures were performed, the correlation between manual and automated quantification of these complex behavioral events markedly increased. Although there were still some discrepancies in the reported values for each endpoint, the overall relationship between experimental groups was preserved, highlighting the need for continued validation and optimization of automated detection settings (see optimized settings in Fig. S2 of Supporting Information). Finally, as an additional (physiological) validation of our findings, whole-body cortisol levels confirmed high- and low- behavioral profiles. All anxiolytic manipulations (Fig. 7) significantly reduced whole body cortisol, whereas all anxiogenic manipulations used here predictably elevated cortisol levels (Fig. 3, also see Fig. S1 in Supporting Information).

## Discussion

Our examination of different novel tank dimensions addressed recently published studies on behavioral tests of anxiety in zebrafish [36], [76], including conflicting findings on bottom dwelling in the open tank tests [56], [77]. Illustrated by 3D swim path reconstructions, we confirmed that this behavioral response is not specific to particular tank types (Fig. 2), supporting the validity of the novel tank test as a model of affective behavior in adult zebrafish (also see [39], [40], [50], [78]).

Overall, this study represents the first large-scale multi-domain analysis of adult zebrafish spontaneous locomotion in novel environments, in which manual and automated behavioral endpoints were precisely integrated for each individual animal. We were also able to rapidly examine the spatiotemporal dynamics of swimming activity, and determine how the video-tracking software detects these events.

The present study utilized a novel approach to video-tracking zebrafish behavior which (unlike previous studies [47], [49], [67], [79], [80], [81], [82], [83]) linked zebrafish locomotion to specific anxiety-related behavior by combining 3D visualization with event-based and manual observation within a single raw track data file for each experimental subject. Although acquiring video-tracking data with the highest possible sampling rate (30 fps) represented a major 3-fold increase from previous studies of zebrafish affective behavior, the ability to reconstruct swimming trajectories for each zebrafish (Fig. 4–6) was a direct result of choosing a high sampling rate. This approach allowed us to visualize the behavior of zebrafish in a manner that has not been performed previously. The 3D swim path reconstructions presented here offer new perspectives to examine zebrafish behavior, since viewing the complete zebrafish swim path within its spatiotemporal context is impossible to perform during manual observation. 3D swim path reconstructions also may lead to the identification and characterization of previously undetectable behavioral endpoints. For example, based on our unpublished observations, proper quantification and characterization of particular movement patterns (e.g., loops, slide and fall, ‘figure-8’s) within their spatiotemporal context (top-left, top-right, bottom-left, bottom-right, first/last 3 min, rapid, slow) seem to be sensitive to specific behavioral profiles (e.g., withdrawal anxiety states, psychological anxiety states, fear/panic-like states or neurological/motor deficits).

Clustering all behavioral endpoints (Fig. 7) also provided valuable, global insights. For example, sub-clusters 1, 2, 4 and 7 reconfirmed the predictive validity of traditional *primary* measures of zebrafish anxiety (Fig. 3). Sub-clusters 2 and 4 provided strong evidence that automated detection of freezing behavior can be achieved with automated movement or mobility parameters, particularly after optimization of acquisition settings (as explained in Tables S3 and S4 of Supporting Information). Sub-cluster 3 showed that alternations in meandering are more pronounced during periods of high anxiety. As previously mentioned, we observed some “noise” in the movement data while reviewing 3D swim path reconstructions. This “noise” emerged as spikes in a few automated endpoints, with meandering most prominently affected. Therefore, a focused examination of this endpoint will be necessary to determine if this overall trend found in anxiogenic treatments is a product of erratic movements or freezing activity, and if meandering holds significant research value in the novel tank test. Finally, sub-cluster 8 highlighted another interesting difference between anxiolytic and anxiogenic responses. In our study, all anxiogenic treatments reduced normal elongations, while anxiolytic treatments increased contracted elongations. Elongation is a measure of surface area or detected body size at a given time, and occur more frequently in fish calmly navigating throughout the tank (e.g., following anxiolytic treatments). While the exact value of these body elongation parameters merit further scrutiny, our results imply that, in general, body shape-based indices may be useful in dissecting high- and low-anxiety states in zebrafish (Fig. 7) most optimal settings (see example and more details in Fig. S2 of Supporting Information).

The ability to integrate manual and automated behavioral data provides novel opportunities to perform in-depth dissection of zebrafish behavior. This study introduces techniques and approaches to perform such analyses, and provided preliminary investigations to this end. Our on-going research seeks to provide more evidence for this by evaluating techniques of *movement pattern analysis* to detect and quantify observed sub-sets of swimming activity. In general, a movement pattern refers to any recognizable spatial/temporal regularity in movement data [47], [80], [84]. In animal research, these movement patterns may emerge as frequent substructures in trajectory data between similar treatment classes (e.g., drugs, genetic or environmental manipulations) or domains (i.e., affective or cognitive states). For example, movement pattern analysis was successfully applied to medaka fish to create accurate predictive models of fish movement based on high-density trajectory data sets [79], [83], [85]. Likewise, applying movement pattern analysis to zebrafish may help identify patterns common for various challenges, formalizing the classifications of these observations into quantitative models.

Increasing the overall data density per zebrafish presented several problems that merit mentioning here. First, global evaluation of the effects of experimental manipulations across all manual, event-based and automated endpoints in a large scale study is difficult using traditional bar or line graphs (see Fig. S1 of the Supplementary Material) and may be markedly improved by 3D swim path reconstructions proposed here (Fig. 2, 4–6). At the same time, while the analysis of raw data set requires additional pre-processing steps within a carefully standardized operation protocol, this amount of work can be significantly alleviated using custom Microsoft Excel macros and/or programming scripts to automate these repetitive formatting tasks. Integrating data into dense track files for each fish, however, strongly supports the use of databases to store and query behavioral data. This ability outweighs the additional pre-processing steps because the available sample size for statistical examination grows with each experimental data set.

This approach may allow for a more sophisticated representation of differences in the “classical” endpoints in relation to their spatiotemporal dynamics following optimization of data as described earlier. The striking overlap between observer- and computer-generated indices in 3D traces (Fig. 5–6) opens the opportunity for further refinement of video-tracking, and may eventually lead to fully automated 3D-based neurophenotyping tools to quantify zebrafish anxiety. In line with this, the sensitivity of zebrafish behavior to both acute (e.g., alarm pheromone, caffeine) and more chronic (e.g., strain-specific anxiety, repeated withdrawal) stressors demonstrated in this study (Fig. 3–7) supports the potential utility of adult zebrafish models to study both state and trait anxiety responses.

Likewise, while our present study focused on anxiety-related responses in adult zebrafish, future research may expand this approach to other behavioral domains, including spatial memory, reward, aggression and sexual behavior. For example, given interesting courting rituals in zebrafish and their rich social and aggressive behaviors [24], [49], [59], [61], [86], [87], [88], 3D-based quantification, mapping and dissection of these domains, based on our approach, may represent a promising direction of research. Furthermore, because of robust differences in zebrafish locomotion following epileptogenic drugs [89], it is likely that our multidimensional approach may be used to assess zebrafish epilepsy-like phenotypes. With the sensitivity and data density of swim track reconstructions, it is possible that computer stimulation of zebrafish anxiety-like behavior can also be developed based on our approach. Empowered by a growing database of experimental data, such intelligent *in-silico* models may find applications in research and teaching.

In conclusion, this study provided a detailed 3D-based approach to phenotyping of zebrafish anxiety-related behaviors, and presented an innovative method for automated visualization and quantification of their swimming activity. The present study further validated the novel tank test as a novelty-based model to analyze zebrafish anxiety, showing that automatic video-tracking systems are both a reliable addition to manual observation, and a tool for a multivariate analysis of zebrafish behavioral endpoints. The use of 3D reconstruction of movement patterns (Fig. 4–6), in combination with event-based behavioral scoring, enabled a more precise deconstruction of zebrafish behavior. As rodent models used in neurobehavioral research are mainly based on 2D movement, zebrafish paradigms offer an enhanced dimensionality of behavioral phenotyping. Therefore, our growing understanding of zebrafish 3D behavior lays an important foundation for neurobehavioral research using these models.

## Materials and Methods

### Ethics Statement

All experimental procedures were in compliance with National and Institutional guidelines on animal experimentation and care.

### Animals and housing

A total of 625 adult (4–7 months) wild-type *short-fin* (n = 612) and *leopard* (n = 13) zebrafish (∼50∶50 male:female ratio) were obtained from a local commercial distributor (see Fig. 3 for details of cohorts and animal sample sizes). All fish were housed in groups of 20–30 per 20 L tank and given at least 10 days to acclimate to the laboratory environment. Tanks were filled with filtered facility water maintained at a temperature of 25–27°C. Illumination was provided via fluorescent light bulbs on a 12 hour cycle (on 6:00 h; off 18:00 h) consistent with the standards of zebrafish care [90]. Fish were fed Tetramin Tropical Flakes (Tetra USA, Blacksburg, VA) twice daily. Behavioral testing was performed between 11:00 and 15:00 h, using treated water (maintained at the same temperature) and experimentally naïve fish. Following observation, animals were euthanized with 500 mg/L Tricaine and held at −80°C for cortisol analysis (as described in [28]). All experimental procedures were in compliance with National and Institutional guidelines on animal experimentation and care.

### Novel tank testing

The standard (small) novel tank apparatus was a 1.5 L trapezoidal tank (15 height ×28 top ×23 bottom ×7 cm width; Aquatic Habitats, Apopka, FL; Fig. 1 and 2) maximally filled with water and divided into two equal virtual horizontal portions, by a line marking the outside walls [28], [39], [71]. The area above this mid-line represented the ‘upper half (top)’ of the novel tank, while the region below represented the ‘lower half (bottom)’ of the novel tank (Fig. 1). In a separate experiment, a large 40 L rectangular tank (60 cm length ×25 cm width ×30 cm height) was used to observe fish behavior (Fig. 2). All apparatuses rested on a level, stable surface.

### Experimental manipulations

To modulate zebrafish anxiety, several genetic, psychological and pharmacological manipulations were used in this study (Fig. 3). A variant genetic strain of zebrafish used here was the leopard strain, shown to display elevated baseline anxiety [28]. Psychological stress was induced by acute alarm pheromone exposure, as described previously [28]. Briefly, individual fish were placed into a 3-L beaker with aquarium water containing 7 ml alarm pheromone solution, for 5 min immediately prior to testing in the novel tank. Pharmacological treatments were performed via immersion of individual fish in 1–3 L of drug-treated filtered facility water. For chronic treatment paradigms, drugs were administered to the hometank with experimental zebrafish daily for 2 weeks; respective controls were housed in identical conditions in absence of the drug. Anxiolytic manipulations included chronic fluoxetine (100 µg/L for 2 weeks) [28], chronic ethanol (0.3% vol/vol for 1 week) [27], [28], [41], [57], [58], and chronic morphine (1.5 mg/L for 2 weeks) [27] treatments, as well as acute nicotine exposure (10 mg/L for 5 min) [39], [40], [78]. Anxiogenic agents included acute caffeine (250 mg/L for 20 min) [28] and repeated withdrawal from chronic morphine treatment, performed as previously described [27]. Briefly, following 1 week of chronic morphine exposure, fish were placed into a 3-L beaker with untreated water for 3 h, twice per day for 1 week, followed by testing in the novel tank test, as described above.

### Behavioral quantification

For each experiment, zebrafish behavior in the 6-min novel tank test was quantified using three different methods: manual observation, event-based scoring and automated video analysis (Fig. 1, Table S1 of Supporting Information). Manual observation was performed ‘live’ by trained observers who recorded behavior immediately after placing the zebrafish into the novel tank. As previously described [28], [56], [58], [71], [91], the following endpoints were recorded: latency (s), transitions (whole-body crossing into upper half) to and time spent (s) in, the upper half of the novel tank, the frequency of erratic movements (sharp changes in direction, high velocity, unorganized darting typically along bottom of tank), as well as the frequency and duration of freezing bouts (total absence of movement, except for eyes and gills, for 2 s or longer). During ‘live’ manual observation, videos (in MPEG1 format) of each trial were recorded via auto-focusing 2.0 MP USB webcams placed approximately 50 cm in front of (side-view) and above (top-view) the novel tanks and connected to laptop computers (Fig. 1).

Event-based scoring and automated video analysis was performed on recorded videos using EthoVision XT7 (Noldus IT, Wageningen, Netherlands). Event-based scoring was performed by a trained observer (typically different from the original ‘live’ observer) in parallel to automated acquisition, by watching the video playback and entering behavioral events using customized keystrokes. A mutually-exclusive behavior group was established to differentiate between states of swimming (“S”), erratic movements (“E”) and freezing (“F”) (see [50] for details). Swimming consisted of normal, continuous motion involving caudal and pectoral fins, while erratic movements and freezing criterion were the same as described above.

Video-tracking was performed in EthoVision XT7 on recorded videos with the maximum sample rate of 30.0 fps. The novel tank arenas were established for each trial (side view) including “top” and “bottom” zones. The origin axes (0,0) were calibrated at the center of the tank in order to standardize spatial coordinates across trials. For top view videos, rectangular arenas were set and the origin axes were placed along the rear (or back) of the tank. Trial Control settings were configured to start acquisition after the subject was detected within the arena for less than 1 s. Detection settings were selected to most accurately acquire zebrafish behavior. Movement tracks were smoothed (across 10 samples) and examined for abnormalities (e.g., missing samples, reflection clustering or rogue points). Trials with widespread abnormalities were reacquired after adjusting arena and detection settings, and standard 2D images of the swim track were generated for all animals tested. Following export and examination of the behavior analysis profile, tracks were interpolated to replace missing values and exported into Excel spreadsheets (see [50], [92] for details).

### 3D Swim Path Reconstructions

For each experiment, raw track data was exported into Excel spreadsheets, pre-processed and formatted to generate 3D swim path reconstructions, as previously described in detail [92], [93]. Briefly, each track was interpolated to replace missing values within the Track Editor of EthoVision XT7. This step replaced missing spatial coordinates by a linear interpolation of the nearest neighbor detection points, or the previous and most recent valid detection coordinates. Raw track files were formatted so that column headers containing independent variables (i.e., “Recording Time”, “X Center”, “Velocity”) were in the first row of the spreadsheet. A “find and replace” procedure was performed to replace null values (“-“) with blank cells. After removing Trial Identification information, track files were renamed to provide this information (i.e., “Control1side.xlsx”). For spatial reconstructions, raw track data from both side and top views was merged using Recording Time, Trial Time and/or unique time stamps within the video (i.e., the fish being placed into the tank) for synchronization, after all preprocessing and formatting steps were performed. Each track file was then saved as a comma separated value (CSV) file and imported into RapidMiner 5.0 software. Each column (Independent Variable) was designated as either a real or integer value-type based on its contents and no special attributes were assigned.

Temporal reconstructions (Fig. 4) were created in a Scatter 3D Color plot, in which X-center, time, and Y- center were attributed to the X,Y- and Z-axes, respectively. Spatial reconstructions (Fig. 5) were generated in a similar manner, with X-center (side-view), X-center (top-view) and Y-center (side-view) plotted on the X,Y- and Z-axes, respectively (see [92] for details). Dependent variables were actively cycled across the path using the color attribute, and tracks were explored using rotation and zooming features. For comparison, axis ranges were standardized, and reconstructions were saved as image files. Representative reconstructions for each experimental manipulation were selected by comparing the complete set of 2D and 3D swim path images, rating from 1 to n based on their similarity to each other (by three observers on a consensus basis) and choosing the middle track as representative (Fig. 6).

### Cluster Analysis

Cluster analysis was applied in this study as an unsupervised statistical method to identify informative subgroups within a large data set [94]. The data used here consists of relative expression values for observed behavioral endpoints, and reflects the intensity in which the experimental group displays a behavior relative to matched controls. Performing this technique, our goal was to identify clusters of experimental manipulations and/or behavioral endpoints based on similarity of their behavioral alterations.

For each experimental manipulation, all behavioral endpoints were preprocessed to obtain the mean and standard deviation for the entire 6-min novel tank test. To standardize expression values of each endpoint, z-score was calculated by subtracting the experimental cohort's mean from the control's mean and dividing by the standard deviation of the controls [16], [94]. The z-score represents the intensity (positive or negative) that experimental fish displayed a behavioral endpoint relative to the control fish of the respective experimental trial. Hierarchical clustering was then performed across behavioral endpoints and experimental manipulations (“arrays”) with Cluster 3.0 (University of Tokyo, Japan), using Spearman Rank Correlation as clustering method, and Average linkage as similarity metric. Clustering results were visualized as a dendogram and colored “array” in Java TrewView (University of Glasgow, UK).

### Statistical Analysis

Behavioral data was analyzed using SPSS 18.0 comparing cohorts with a Mann-Whitney U-test, and with a Kurskal-Wallis test for comparing data across the 6-min test trials. One- or two-way ANOVA, followed by post-hoc Tukey test, was used to analyze the effects of tank type and test time on fish behavior (Fig. 2). Data were not corrected for multiple hypothesis testing in this study. Significance was set at p<0.05 in all experiments.

## Supporting Information

Figure S1 **Original behavioral data for experimental manipulations summarized in** **Fig. 3** **of the manuscript.** Analysis of traditional endpoints shows that anxiogenic (panel a) and anxiolytic (panel b) treatments significantly affect the behavior of adult zebrafish in standardized 6-min novel tank tests. Additionally, high- and low-anxiety-like behavioral profiles are paralleled by respective increases or decreases in whole-body cortisol levels, with the exception of nicotine (which reproducibly elevated cortisol despite observed anxiolytic-like behaviors). Although movement parameters obtained with automated video-tracking techniques are not shown here, datasets presented illustrate the limitations of bar and line plots, also demonstrating the need for novel approaches to globally evaluate zebrafish behavior across multiple experimental designs (some material from this supplementary figure has been published previously in [28], [50]). Data presented as mean ± S.E.M, *p<0.05, **p<0.01, ***p<0.001, # p = 0.05-0.085, trend (U-test).
(PDF)

Figure S2 **Optimization of behavioral data based on computer-generated values and their global analysis using our approach.** Erratic movements and freezing bouts are used here as examples.
(DOCX)

Table S1 **A comprehensive catalogue of traditional (manual) and automated (EthoVision XT7-generated) behavioral parameters characterizing adult zebrafish behavior in the novel tank test (see** [**50**] **for details).**
(DOCX)

Table S2 **Optimization of automated movement parameters to improve correlation with observed behavioral states (also see Fig. S2 for examples).** Raw track data (used here to generate 3D swim path reconstructions in fish presented in Fig. 6) consists of exact expression values of automatically-generated movement parameters. Precisely integrating manually observed and event-based behavioral records within this temporal dataset allowed us to examine how automated movement endpoints change during specific behavioral events (e.g., Swimming, Erratic Movement or Freezing), see Fig. S2 for details. Briefly, from each anxiolytic or anxiogenic experimental manipulation, raw individual track data was compiled into a single, very large dataset and categorized based on manually registered behavioral events (see above). Average values were then obtained for movement parameters of interest during that behavioral state to reflect the ways in which automated video-tracking software detect changes in locomotion during specific behaviors of interest. These optimized averaged values were applied to all fish used in this study, leading to a marked improvement of correlation between manual vs. computer generated endpoints. Overall, two key observations can be made here: 1) velocity ranges for each behavioral event and 2) averaging intervals across samples is efficient to reduce low velocity noise. With this information, Movement and Mobility analysis profiles were refined to optimize detection of complex behavioral events (Fig. S2; data presented as mean ± S.E.M). Note that while the exact values of manual vs. computer-based analyses were not the same, they correlated strongly between each other for specific behaviors examined here.
(DOCX)

Table S3 **Correlation analysis of manual, event-based and automated behavioral quantification techniques on various endpoints assessed for** ***all*** **fish used in the present study.** Our study integrated three approaches to quantify zebrafish behavior: manual observation (performed during the novel tank test), event-based scoring (performed by trained observers during acquisition) and automated video-tracking of zebrafish movement (See Materials and Methods for details). To determine the reliability of these methods, Spearman correlation coefficients for presented endpoints were calculated between each quantification method. This table compares manual observation to automated quantification of traditional novel tank endpoints (Latency to upper half, Transitions to upper half and Time spent in upper half). Across several experimental trials, there was a significantly high correlation (most above 90%) between these techniques, illustrating the reliability of video-tracking tools in zebrafish behavioral research. This table also presents correlations between manually observed and event-based scoring of more complex behavioral responses (erratic movements, freezing bouts and duration), assessing consistency to characterize zebrafish behavior in front of the novel tank and subsequent event-based scoring of videos from the same experiment. Overall, there was a strong correlation between these approaches (most above 70%), although some inconsistencies (particularly in regards to erratic movement) may arise from the fact that manual observation is prone to subjective variations, requiring more objective automated approaches, such as presented here.
(DOCX)

Table S4 **Spearman correlation coefficients generated by unsupervised, hierarchical cluster analysis.** The cluster analysis performed in our study resulted in several meaningful sub-groups of related experimental treatments or behavioral endpoints. With a correlation coefficient of 0.652, anxiogenic treatments grouped within Cluster I, whereas strongly correlated (r_s_ = 0.940) anxiolytic treatments formed the basis of Cluster II. The gathering of manual, event-based and automated endpoints within highly correlated sub-clusters 4 and 5, strongly illustrates the similarities in which these methods quantify related behavioral events.
(DOCX)
