# Supplementary material for: Three-Dimensional Neurophenotyping of Adult Zebrafish Behavior
Source: PLoS One. 2011 Mar 7;6(3):e17597. doi: 10.1371/journal.pone.0017597 (PMC3049776; doi:10.1371/journal.pone.0017597)

Figure S1a. Anxiogenic experimental manipulations

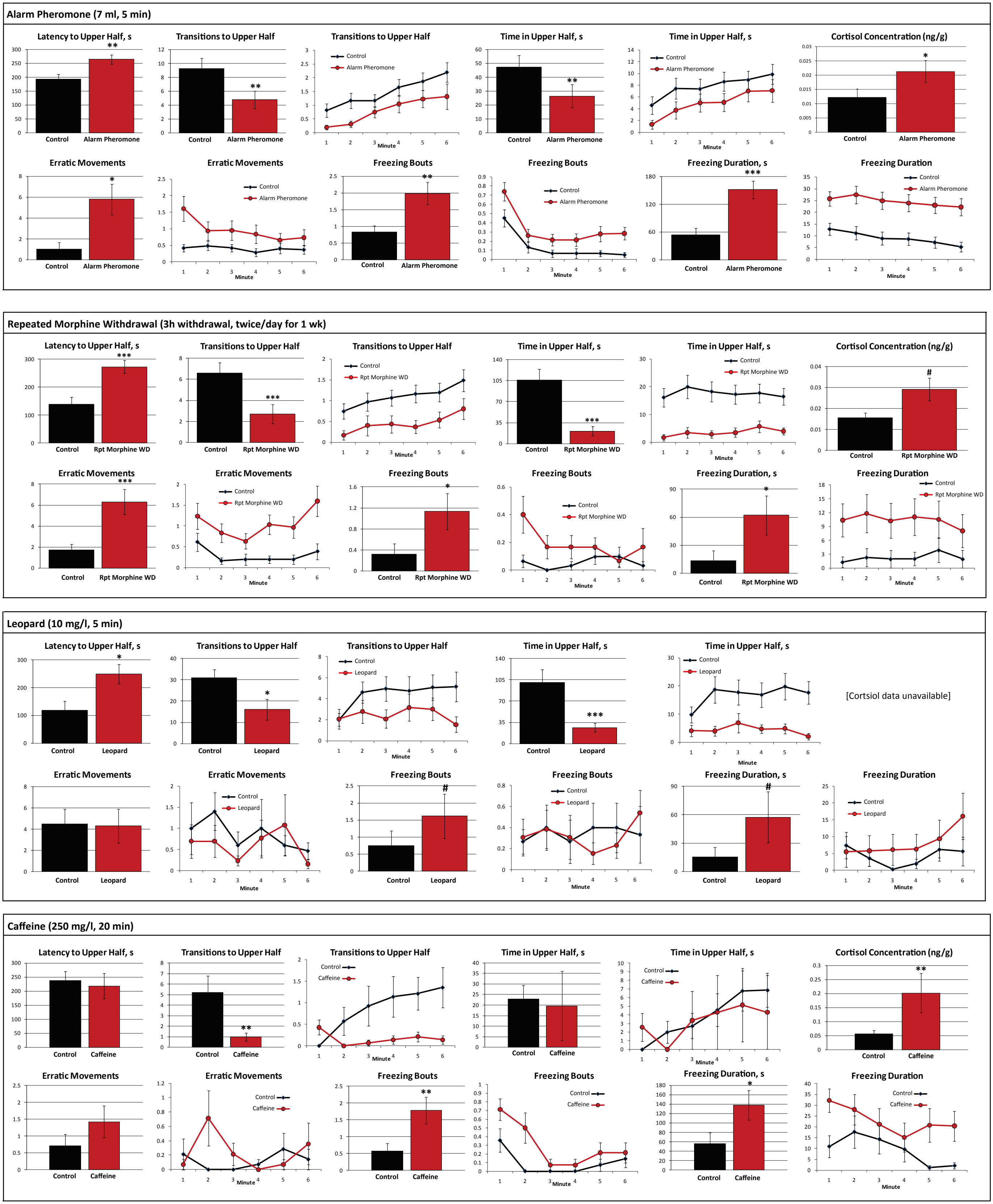

Figure S1b. Anxiolytic experimental manipulations

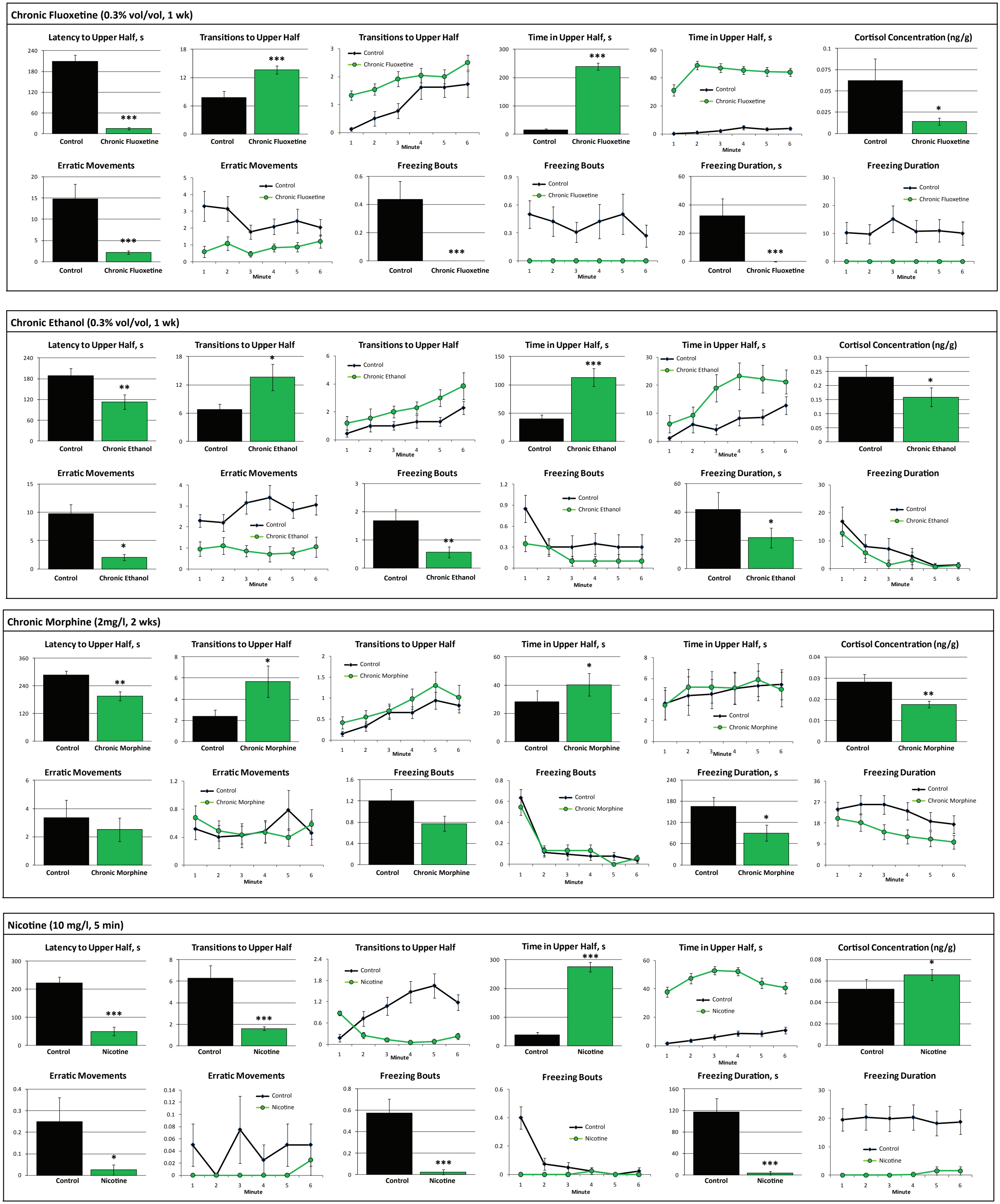

Supplement: Figure S1 — Original behavioral data for experimental manipulations summarized in Fig. 3 of the manuscript. Analysis of traditional endpoints shows that anxiogenic (panel a) and anxiolytic (panel b) treatments significantly affect the behavior of adult zebrafish in standardized 6-min novel tank tests. Additionally, high- and low-anxiety-like behavioral profiles are paralleled by respective increases or decreases in whole-body cortisol levels, with the exception of nicotine (which reproducibly elevated cortisol despite observed anxiolytic-like behaviors). Although movement parameters obtained with automated video-tracking techniques are not shown here, datasets presented illustrate the limitations of bar and line plots, also demonstrating the need for novel approaches to globally evaluate zebrafish behavior across multiple experimental designs (some material from this supplementary figure has been published previously in [28], [50]). Data presented as mean ± S.E.M, *p<0.05, **p<0.01, ***p<0.001, # p = 0.05-0.085, trend (U-test). (PDF) [file pone.0017597.s001.pdf]
