# Supplementary material for: Three-Dimensional Neurophenotyping of Adult Zebrafish Behavior
Source: PLoS One. 2011 Mar 7;6(3):e17597. doi: 10.1371/journal.pone.0017597 (PMC3049776; doi:10.1371/journal.pone.0017597)
Supplement: Figure S2 — Optimization of behavioral data based on computer-generated values and their global analysis using our approach. Erratic movements and freezing bouts are used here as examples. (DOCX) [file pone.0017597.s002.docx]

| Swimming | **Automated Movement Parameter** | | | | | |
| --- | --- | --- | --- | --- | --- | --- |
|  | Distance Traveled | Velocity | TurnAngle | TurningRate | TurnBias | Meander |
| Control | 0.00153 | 0.04597 | 0.02684 | 148.10615 | 0.80439 | 21414.63256 |
| Anxiogenic | 0.00107 | 0.03213 | 0.07761 | 121.14408 | 2.32617 | 37195.89932 |
| Anxiolytic | 0.00106 | 0.03188 | 0.25088 | 112.71234 | 7.51871 | 22116.34120 |
| **Overall Average** | **0.00122** | **0.03666** | **0.11844** | **127.32086** | **3.54975** | **26908.95769** |
| Erratic Movement | **Automated Movement Parameter** | | | | | |
|  | Distance Traveled | Velocity | TurnAngle | TurningRate | TurnBias | Meander |
| Control | 0.00340 | 0.10189 | 5.02998 | 201.27452 | 150.74839 | 2287.62557 |
| Anxiogenic | 0.00226 | 0.06771 | 3.02550 | 202.34916 | 90.67441 | 30399.78513 |
| Anxiolytic | 0.00165 | 0.04932 | 0.86541 | 104.12243 | 25.93628 | 5819.20485 |
| **Overall Average** | **0.00243** | **0.07297** | **2.97363** | **169.24871** | **89.11970** | **12835.53852** |
| Freezing | **Automated Movement Parameter** | | | | | |
|  | Distance Traveled | Velocity | TurnAngle | TurningRate | TurnBias | Meander |
| Control | 0.00001 | 0.00037 | -0.14327 | 648.18234 | -4.29418 | 8912561.10062 |
| Anxiogenic | 0.00008 | 0.00247 | 0.33190 | 734.46848 | 9.94699 | 6028634.96476 |
| **Overall Average** | **0.00005** | **0.00142** | **0.09431** | **691.32541** | **2.82641** | **7470598.03269** |
| **Observations:** 1) The greatest changes in distance, velocity, turn angle and turn bias occur during erratic movements. 2) Meandering and Turning Rate are sharply increased while the zebrafish is reportedly freezing. | | | | | | |
| **Interpretation:** While Swimming, velocity approximately ranges between 0.032–0.046 m/s. During Erratic Movements, velocity ranges between 0.049–0.102 m/s. If freezing, velocity is expected to be within 0.0004–0.0025 m/s. Given the calculation of these endpoints incorporates distance moved between sequential time points, observed increases in Turning Rate and Meandering parameters must be the result of ‘noise’ that is a by-product of using a high sampling rate. In particular, inconsequential movements arising from minor fluctuations in subject detection that occur during extremely small changes in location cause Turning Rate and Meandering to spike. To correct for these effects, an averaging interval should be used on respective movement parameters. | | | | | | |
| **Application:** EthoVision XT7 offers discrete Movement and Mobility parameters that can be customized to quantify activity above and/or below set velocity or spatial displacement thresholds. Using ranges listed above, we were able to quickly optimize movement and mobility parameters to increase the correlation of these endpoints with those quantified with manual, even-based scoring (see examples in this table further). ***Rapid Movement*** detects movement activity between 0.1 and 0.04 m/s, averaged over 10 s, as suggested to occur during Erratic Movements above. ***Slow Movement*** detects movement activity between 0.002 and 0.0003 m/s, averaged over 10 s, as suggested to occur during Freezing above. ***Mobility*** default settings detect high mobility as > 80% spatial displacement, immobility as spatial displacements < 20%, and do not average over sample. After comparing several variations, we found the most optimal mobility thresholds to be > 75% and < 3%, with a 10-sample averaging interval. | | | | | | |
