# Supplementary material for: Three-Dimensional Neurophenotyping of Adult Zebrafish Behavior
Source: PLoS One. 2011 Mar 7;6(3):e17597. doi: 10.1371/journal.pone.0017597 (PMC3049776; doi:10.1371/journal.pone.0017597)
Supplement: Table S1 — A comprehensive catalogue of traditional (manual) and automated (EthoVision XT7-generated) behavioral parameters characterizing adult zebrafish behavior in the novel tank test (see [50] for details). (DOCX) [file pone.0017597.s003.docx]

**Table S1**

| **Behavioral index (unit)** | **Definition** | **Interpretation** | **Quantification methods *** |
| --- | --- | --- | --- |
| ***Primary endpoints (also see Fig. 3)*** | | | |
| Latency to top (s) | Time until the first transition into the upper half of the novel tank | Longer latency suggests increased anxiety-like state | Manual, Automated |
| Transitions to top | Number of crosses from bottom to top | More transitions indicate lower anxiety levels | Manual, Automated |
| Time in top (s) | Total time spent in defined top portion | Longer duration suggests lower anxiety/fear | Manual, Automated |
| Erratic movements | Sharp changes in direction and/or velocity (rapid darting) | More erratic swimming indicates increased anxiety-like state | Manual |
| Freezing bouts | Total absence of movement (except for eyes and gills) for 2 s or longer | More freezing bouts indicate increased anxiety/fear | Manual |
| Freezing duration (s) | Total time spent frozen | Longer freezing indicate elevated anxiety | Manual |
| ***Additional endpoints*** | | | |
| Distance traveled (m) | Total distance traveled in the novel tank | Reflects locomotion functioning | Automated |
| Velocity (m/s) | Average velocity during trial | Reflects locomotion functioning | Automated |
| Turn angle (°) | Absolute change in direction of subject between two samples | Helpful for detecting stereotypic movements | Automated |
| Turning rate (°/s) | Absolute change in direction of movement between two consecutive samples | Reflects amount of turning per unit time (high values suggest local searching behaviors) | Automated |
| Turn bias (°/s) | Relative change in direction of body between two samples | Expresses the speed of change in direction of movement, reflects circular tendencies in behavior | Automated |
| Meander (°/m) | Absolute change in direction of movement per distance moved | Expresses overall turning rates and patterns | Automated |
| Movement  (moving/not moving) | Discrete variable based on average velocity and defined thresholds (0.017-0.02 m/s) | Reflects overall locomotor activity | Automated |
| Elongation (stretched, normal or contracted) | Average of subject’s pixels from 0% (circle) to 100% (line) against defined 90%-35% thresholds | Objective assessment of frequency and duration of various postures | Automated |
| Mobility (highly mobile, mobile or immobile) | Change in subject’s pixels between two samples (relative to defined 80%-20% thresholds) | Expresses degree of movement independent of spatial displacement | Automated |

*Potential quantification methods that both can be applied to quantify the respective endpoints [50].
