# Supplementary material for: Three-Dimensional Neurophenotyping of Adult Zebrafish Behavior
Source: PLoS One. 2011 Mar 7;6(3):e17597. doi: 10.1371/journal.pone.0017597 (PMC3049776; doi:10.1371/journal.pone.0017597)
Supplement: Table S3 — Correlation analysis of manual, event-based and automated behavioral quantification techniques on various endpoints assessed for all fish used in the present study. Our study integrated three approaches to quantify zebrafish behavior: manual observation (performed during the novel tank test), event-based scoring (performed by trained observers during acquisition) and automated video-tracking of zebrafish movement (See Materials and Methods for details). To determine the reliability of these methods, Spearman correlation coefficients for presented endpoints were calculated between each quantification method. This table compares manual observation to automated quantification of traditional novel tank endpoints (Latency to upper half, Transitions to upper half and Time spent in upper half). Across several experimental trials, there was a significantly high correlation (most above 90%) between these techniques, illustrating the reliability of video-tracking tools in zebrafish behavioral research. This table also presents correlations between manually observed and event-based scoring of more complex behavioral responses (erratic movements, freezing bouts and duration), assessing consistency to characterize zebrafish behavior in front of the novel tank and subsequent event-based scoring of videos from the same experiment. Overall, there was a strong correlation between these approaches (most above 70%), although some inconsistencies (particularly in regards to erratic movement) may arise from the fact that manual observation is prone to subjective variations, requiring more objective automated approaches, such as presented here. (DOCX) [file pone.0017597.s005.docx]

| **Treatment** | | **Traditional Novel Tank Test Endpoints** | | |
| --- | --- | --- | --- | --- |
|  |  | **Latency to Upper Half, s*** | **Transitions to Upper Half*** | **Time Spent in Upper Half, s*** |
| Alarm Pheromone | | 0.974 | 0.965 | 0.964 |
| Rpt Morphine WD | | 0.919 | 0.965 | 0.978 |
| Acute Caffeine | | 0.749 | 0.692 | 0.786 |
| Chronic Fluoxetine | | 0.934 | 0.775 | 0.923 |
| Chronic Ethanol | | 0.702 | 0.917 | 0.834 |
| Chronic Morphine | | 0.922 | 0.912 | 0.935 |
| Acute Nicotine | | 0.984 | 0.934 | 0.989 |
|  |  | *All Spearman correlation coefficients were statistically significant with p < 0.001. | | |

| **Treatment** | | **Complex Behavioral Responses** | | |
| --- | --- | --- | --- | --- |
|  |  | **Erratic Movement*** | **Freezing Bouts*** | **Freezing Duration, s*** |
| Alarm Pheromone | | 0.759 | 0.498 (p = 0.025) | 0.834 |
| Rpt Morphine WD | | 0.569 (p = 0.002) | 0.753 | 0.877 |
| Acute Caffeine | | 0.202 (ns, p = 0.302) | 0.735 | 0.788 |
| Chronic Fluoxetine | | 0.102 (p = 0.669) | 0.371 (p = 0.107) | 0.534 (p = 0.015) |
| Chronic Morphine | | 0.192 (p = 0.338) | 0.623 | 0.701 |
|  |  | *All Spearman correlation coefficients were statistically significant with p < 0.001. | | |
