# Supplementary material for: Three-Dimensional Neurophenotyping of Adult Zebrafish Behavior
Source: PLoS One. 2011 Mar 7;6(3):e17597. doi: 10.1371/journal.pone.0017597 (PMC3049776; doi:10.1371/journal.pone.0017597)
Supplement: Table S4 — Spearman correlation coefficients generated by unsupervised, hierarchical cluster analysis. The cluster analysis performed in our study resulted in several meaningful sub-groups of related experimental treatments or behavioral endpoints. With a correlation coefficient of 0.652, anxiogenic treatments grouped within Cluster I, whereas strongly correlated (rs = 0.940) anxiolytic treatments formed the basis of Cluster II. The gathering of manual, event-based and automated endpoints within highly correlated sub-clusters 4 and 5, strongly illustrates the similarities in which these methods quantify related behavioral events. (DOCX) [file pone.0017597.s006.docx]

| **Spearman Correlation Coefficients of Cluster Analysis** | | |
| --- | --- | --- |
|  |  |  |
| **Cluster I** | Anxiogenic Treatments | **0.652** |
| **Cluster II** | Anxiolytic Treatments | **0.94** |
|  | **Behavioral Cluster A** |  |
| **Sub-cluster 2** | (Turn Angle, Turning Rate, Slow Movements, Freezing Duration, Immobile Duration, Slow Movement Duration) | **0.892** |
| **Sub-cluster 4** | (Freezing Bouts, Immobile Bouts, Mobile Bouts) | **0.703** |
| **Sub-cluster 5** | (Erratic Movements, Stretched Duration) | **0.564** |
|  | **Behavioral Cluster B** |  |
| **Sub-cluster 6** | (Rapid Movement, Distance Traveled, Velocity, Rapid Movement Duration, Highly Mobile Duration, Contracted Duration) | **0.562** |
| **Sub-cluster 8** | (Time in Upper Half, Contracted Body Bouts, Highly Mobile Bouts, Mobile Duration, Normal Body Bouts, Stretched Body Bouts) | **0.511** |
| **Sub-cluster 9** | (Turn Bias, Normal Body Duration) | **0.442** |
